# Evolution, development, and organization of the cortical connectome

**DOI:** 10.1371/journal.pbio.3000259

**Published:** 2019-05-10

**Authors:** Miguel Ángel García-Cabezas, Basilis Zikopoulos

**Affiliations:** 1 Neural Systems Laboratory, Department of Health Sciences, Boston University, Boston, Massachusetts, United States of America; 2 Human Systems Neuroscience Laboratory, Department of Health Sciences, Boston University, Boston, Massachusetts, United States of America; 3 Department of Anatomy and Neurobiology, Boston University School of Medicine, Boston, Massachusetts, United States of America; 4 Graduate Program in Neuroscience, Boston University, Boston, Massachusetts, United States of America

## Abstract

Hypotheses and theoretical frameworks are needed to organize and interpret the wealth of data on the organization of cortical networks in humans and animals in the light of development, evolution, and selective vulnerability to pathology. Goulas and colleagues compared several hypotheses of cortical network organization in 4 mammalian species and conclude that (1) the laminar pattern of cortico-cortical connections is better predicted by the Structural Model, which relates cytoarchitectonic differences of cortical areas to their interconnectedness, and (2) the existence of cortico-cortical connections is related to cytoarchitectonic differences and the physical distance between cortical areas. The predictions of the Structural Model can be applied to the human cortex, in which invasive studies are precluded. Goulas and colleagues advance interesting questions regarding the emergence of cortical structure and networks in development and evolution. Validated theories of cortical structure, development, and function can guide studies of cortical networks likely affected in neurodevelopmental disorders.

## Abstract 2

This Primer explores recent research into the common organizational principles and specializations of the cerebral cortex; these reflect convergence and divergence of evolutionary and developmental mechanisms, and form a basis for understanding cortical function and pathology.

Mapping cortical structure and connections is needed to understand brain function and pathology. Noninvasive tractography and imaging play a major role in advancing our understanding of the typical and atypical development, organization, and function of major cortical networks in humans. However, to identify key features of cortical pathways like laminar patterns and cellular interactions at the synapse level, we rely on invasive neuroanatomical tract-tracing studies in animals. This raises the question of whether or not we can use the wealth of detailed information on the structure and connections of the cerebral cortex in mammals to understand cortical pathways in humans and their disruption in disease. To answer this question, we must identify fundamental principles of cortical structure and network organization that are common across mammals. It is also important to pinpoint species-specific specializations that differentiate humans from other mammals. Collectively, common organizational principles and specializations of the cerebral cortex reflect convergence and divergence of evolutionary and developmental mechanisms and form the basis to understand cortical function and pathology.

## Sensory and motor processing in the cerebral cortex is hierarchical

The cerebral cortex is at the highest level in the hierarchies of neural pathways. Pathways of all internal or external sensory modalities originate in peripheral receptors and ascend in the central nervous system through successive levels to reach the cerebral cortex. In the reverse direction, commands for effectors descend from the cortex to the motor nuclei in the brainstem and the spinal cord (motor actions) and the hypothalamus (autonomic responses and neuroendocrine regulation) [1].

Neural processing within the cerebral cortex is also hierarchically organized. Sensory pathways of each modality access the cerebral cortex through primary areas, which are the first and lowest level in cortical hierarchies. Up one level, there are unimodal association areas that elaborate information from diverse feature-sets within one sensory modality. Pathways from unimodal association areas converge in multimodal association areas and, finally, in limbic areas, the less specialized cortical areas at the last and highest level in cortical hierarchies [2–4]. The flow of sensory information from primary areas all the way up to limbic areas is feedforward. Pathways connecting cortical areas in the opposite direction (from limbic to primary sensory or primary motor through multimodal and unimodal association areas) are feedback pathways [3]. For instance, in the framework of predictive coding [5], predictions flow back from higher to lower areas in the processing hierarchy via feedback pathways [6]. Moreover, motor commands likely originate in cingulate motor limbic areas and reach the primary motor cortex via feedback pathways [7].

## Laminar patterns of connections characterize pathways across cortical hierarchies

A major structural feature of the cerebral cortex is the arrangement of cortical neurons in layers. Pathways connecting cortical areas originate and end in different layers showing different laminar patterns of connections. Feedforward pathways originate in neurons in the supragranular layers II–III and target middle and infragranular layers. Feedback pathways originate in infragranular layers V–VI and target mostly superficial layers I–III. Lateral connections between cortical areas at the same level of the cortical hierarchy originate in supragranular layers II–III and infragranular layers V–VI and terminate in all layers [8].

The intricate network of cortical connections forms the neural basis for most aspects of flexible behavior like cognition, emotion, attention, memory, decision making, action, and consciousness (e. g., [4,6,9]). Is it possible to decipher the unifying principles that underlie the presence or absence of connections between cortical areas and their laminar pattern? Can neuroscientists sketch out a blueprint of the cortical organization applicable to all mammals including humans?

## Cortical connections are predicted by differences in laminar structure of cortical areas

A number of recent studies have used available cortical parcellation schemes and connectivity data sets to address the questions posed above (e.g., [10,11]). In their study reported in this issue of *PLoS Biology*, Goulas and colleagues [12] search for organizational principles linking structure and connections in the cerebral cortex of mice, cats, marmoset monkeys, and macaque monkeys. They compared several hypotheses regarding the presence or absence of connections and the systematic shift of the laminar origin of connections from predominantly infragranular (feedback) to predominantly supragranular (feedforward). One hypothesis relates physical distance between cortical areas to the laminar pattern and the presence of connections [13]. Another hypothesis postulates the organization of laminar patterns of cortical pathways along the rostrocaudal axis of the adult brain [14]. A third hypothesis relates presence, strength, and laminar pattern of connections between cortical areas to gradients of laminar elaboration across the cortex in a relational model called the Structural Model. According to this model, pathways from areas of simpler cytoarchitecture to areas with more elaborate cytoarchitecture originate in infragranular layers V–VI and terminate in supragranular layers II–III (feedback). In the reverse direction, pathways from areas of more elaborate cytoarchitecture to areas of simpler cytoarchitecture originate in supragranular layers II–III and terminate in middle and infragranular layers (feedforward). Pathways connecting areas of comparable cytoarchitecture originate in infragranular and supragranular layers—except layer I—and terminate in all layers (lateral) [15,16].

After comparing these theoretical frameworks Goulas and colleagues [12] show that (1) in cats and macaque monkeys, but not in mice and marmoset monkeys, the presence of connections between cortical areas is related to similarity in laminar elaboration of the connected areas more than to physical distance between these areas; (2) similarities and differences in the laminar elaboration of cortical areas are more closely linked to the laminar origin of connections between 2 areas than their physical position across the rostrocaudal axis of the brain; (3) the relationship between laminar elaboration of cortical areas and connections can predict the laminar pattern of connections in the human cortex, in which invasive studies are precluded; (4) cortical networks in mice, cats, marmoset monkeys, and macaque monkeys include a core of widely connected hubs; (5) the network core is displaced towards areas with simpler laminar elaboration in cats and macaques but not in mice and marmosets.

Goulas and colleagues [12] add to other studies [17–20] that confirm the predictions of the Structural Model over other hypotheses and open new venues that can guide future research on cortical development, evolution, and selective vulnerability to pathology, discussed below.

## Cellular and architectonic features as proxies of laminar elaboration

As outlined above, the Structural Model relates laminar patterns of connections to the laminar elaboration of the connected areas. Cortical areas, each with characteristic architecture, can be sorted into a few cortical types according to the elaboration of their laminar structure. Laminar architecture varies gradually and systematically across the cerebral cortex dividing the cortical landscape into a minimum of 3 major cortical types that are present in different proportions across mammalian species: agranular if they lack the granular cell layer IV, dysgranular if they have an incipient granular layer IV, and eulaminate areas if they have 6 well-developed layers [16,20].

Several architectonic features are correlated, in part, with cortical type. For instance, neuron density, the content of intracortical myelin, or the expression of the nonphosphorylated intermediate neurofilament protein Sternberger-Meyer monoclonal antibody to a nonphosphorylated epitope on human NF-M and NF-H subunits of neurofilament protein (SMI-32) increase, in general, but not always, with laminar elaboration [7,9,16,21]. Thus, these features should be used cautiously as proxies for cortical type. This is relevant for the interpretation of interspecies differences found by Goulas and colleagues [12], because they used neuron density as a proxy for laminar elaboration. The cerebral cortex of primates, including the marmoset, shows further laminar elaboration along cortical gradients than rodents [22], but Goulas and colleagues [12] found that the relationship of the cortical connectome to cortical structure in the marmoset resembles this relationship in the mouse. Future studies in the marmoset could use cortical type analysis or a combination of cellular features related to laminar elaboration, like intracortical myelin and SMI-32 expression. These studies may support the existence of species-specific differences found in the marmoset brain or, alternatively, show that the relationship of the cortical connectome to cortical structure in the marmoset is more similar to the macaque than to the mouse.

## How does the cortical connectome emerge in development and evolution?

**Fig 1:**
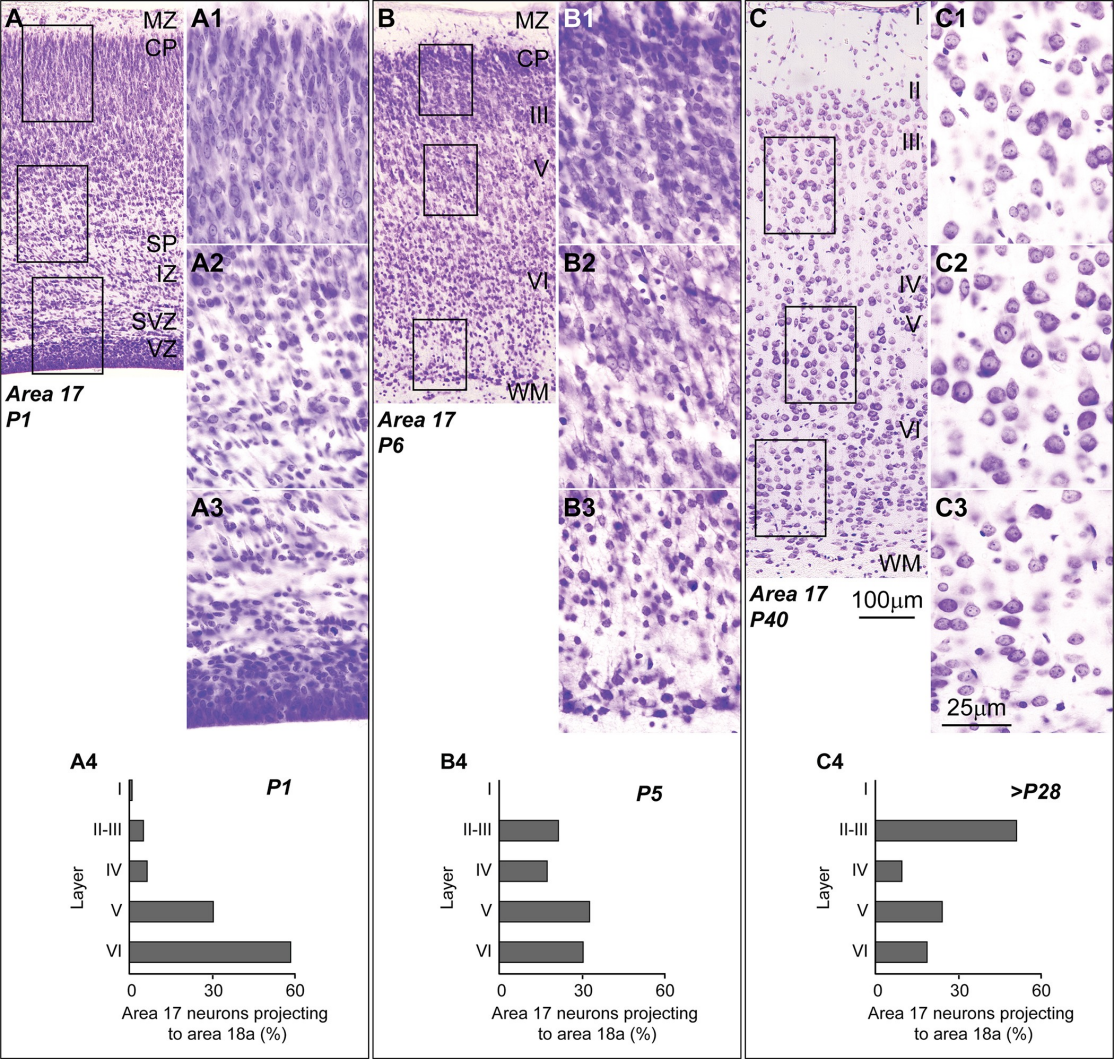
Axons of cortico-cortical pathways arrive at their targets during development in the order of neuron generation (inside-out). (A) Section of area 17 of the rat at P1 (Nissl staining) shows immature neurons in the superficial and middle part of the CP (A1); neurons in the deep part of the CP show better cytological differentiation (A2); The VZ and SVZ are dense, and abundant cells are migrating through the IZ (A3). At P1, neurons in area 17 that project to area 18a are found mostly in infragranular layers V–VI (A4). (B) Section of area 17 of the rat at P6 (Nissl staining) shows immature neurons in the superficial part of the CP (B1); neurons in the middle part of the CP (B2) show better cytological differentiation; neurons in the deep part of the CP (B3) are differentiated and separated by neuropil. At P5, neurons in area 17 that project to area 18a are found in infragranular layers V–VI but also in supragranular layers II–III (B4). (C) Section of area 17 of the rat at P40 (Nissl staining) shows mature neurons across all cortical layers (C1, C2, C3). After P28, neurons in area 17 that project to area 18a are found mostly in supragranular layers II–III, the typical pattern of the adult cortex (C4). Roman numerals indicate cortical layers. Calibration bar in Panel C applies to Panels A, B, and C. Calibration bar in Panel C3 applies to Panels A1–A3, B1–B3, C1–C3. (Note: Panels A, B, C and A1–A3, B1–B3, C1–C3 are an examination of material from a gift of Dr. Alan Peters; data shown in Panels A4, B4, and C4 are from [25]). CP, cortical plate; IZ, intermediate zone; MZ, marginal zone; P1, postnatal day 1; P5, postnatal day 5; P6, postnatal day 6; P28, postnatal day 28; P40, postnatal day 40; SP, subplate; SVZ, subventricular zone; VZ, ventricular zone; WM, white matter.

An intriguing question discussed by Goulas and colleagues [12] is the developmental origin of cortical gradients of laminar elaboration and the patterns of connections linked to these gradients. Data form studies in nonhuman primates show that the time-course of neurogenesis and migration of cortical neurons parallels laminar elaboration [7,16,23]. Areas with simpler laminar architecture develop before areas of more elaborated laminar architecture; therefore, areas with comparable laminar elaboration show comparable neurogenetic time-courses and are more likely to be connected: “what develops together, wires together” [12]. White matter tracts that connect limbic areas, like the cingulate bundle, develop earlier than tracts connecting eulaminate areas [24]. The connections between primary and secondary visual areas of the rat also reach their cortical targets in the order the neurons were generated, following an inside-out pattern from deep to more superficial layers (Fig 1; [25]).

**Fig 2:**
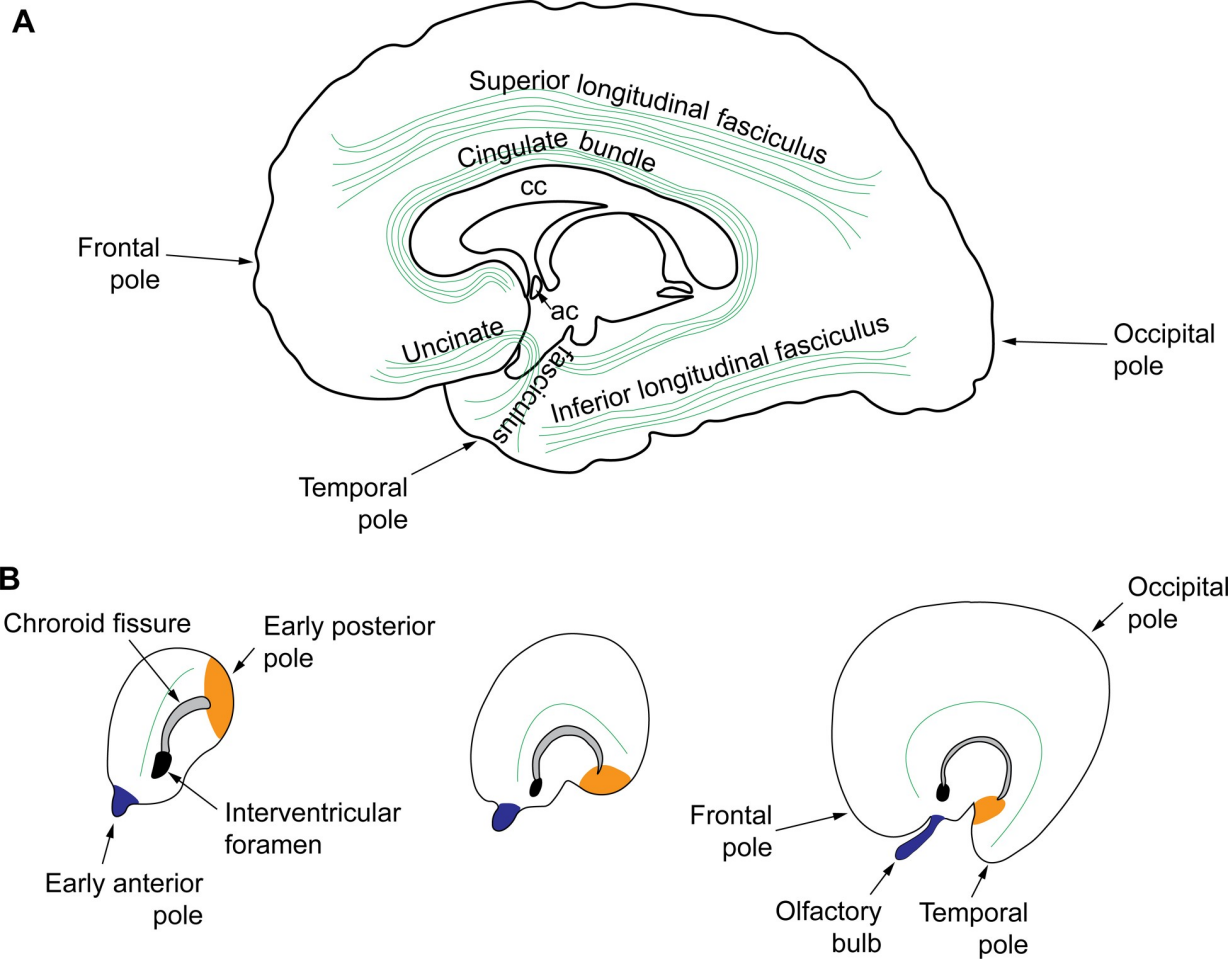
Pathways connecting limbic areas form earlier in development. (A) Sketch of the right hemisphere (medial view) showing the major white matter tracts (green lines) in the human brain (redrawn from Fig 751 in [27]). Tracts connecting limbic areas (cingulate bundle and uncinate fasciculus) are curved and tracts connecting eulaminate areas (superior and inferior longitudinal fasciculi) are straight. (B) Formation of the temporal, occipital, and frontal poles in development (redrawn from Fig 11.4 in [1]). Early in development (left panel), there is an early posterior pole (orange). As the neocortex expands (middle panel), the early posterior pole is pushed anteriorly and ventrally to its final location; the temporal pole is composed of areas that are displaced with the early posterior pole (right panel). We propose that the axons of the cingulate bundle (green line in the three panels) and the uncinate fasciculus arrive to the subplate of their target areas early in development and are curved by the displacement of the early posterior pole to its final location. ac, anterior commissure; cc: corpus callosum.

In this context, the shape of major white matter pathways in the human brain may give us additional clues about the development of cortical networks. It is interesting to note that the cingulate bundle and the uncinate fasciculus that connect limbic areas across lobes are curved. In contrast, the superior and inferior longitudinal fasciculi connect eulaminate areas and are straight (Fig 2A). Based on the shape of these white matter fiber tracts in the adult brain and on the principle “what develops together, wires together,” we suggest that the bent cingulate and uncinate bundles are formed during development before the straight superior and inferior longitudinal fasciculi. At an early prenatal stage, when the prospective temporal pole is close to the early posterior pole of the telencephalon, the cortical plate is likely formed only by prospective limbic areas that connect through straight prospective cingulate and uncinate bundles. The later expansion of eulaminate areas and their pathways, which take longer to develop, pushes the temporal pole to its final location and bends the cingulate and uncinate bundles that acquire their final shape (Fig 2B). The superior and inferior longitudinal fasciculi likely form straight when the temporal pole is in its final location and keep this shape in the adult brain. The proximity during development of cortical areas of similar laminar elaboration also explains why areas that in the adult brain are far apart are strongly connected, like anterior cingulate area 25 and area prostriata in the occipital lobe [26], both of which are limbic [3]. Future developmental tract tracing studies in nonhuman primates could test this hypothesis and describe differences in the time-course of development for different cortico-cortical pathways.

**Fig 3:**
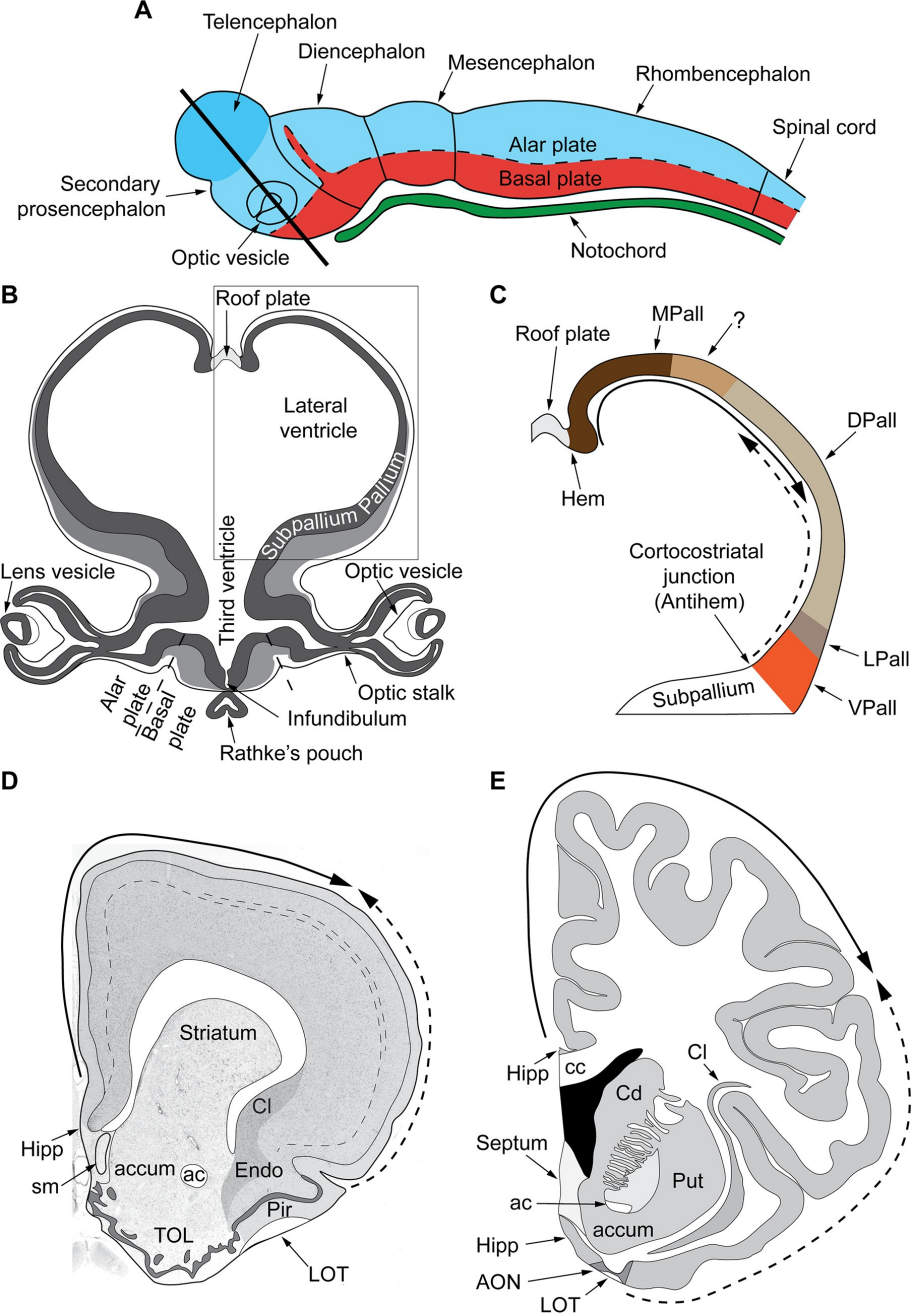
A mechanism for the expansion of the neocortex in development and evolution. (A) Sketch of the mammalian neural tube showing the proneuromeric compartments according to [1]. The alar plate is colored in blue and the basal plate is red; the notochord is green. The telencephalic vesicle (highlighted in darker blue) grows out of the alar plate of the secondary prosencephalon. (B) Section through the secondary prosencephalon at the level of the black line in (A) shows the optic vesicles and the telencephalic vesicles (redrawn from Fig 264 in [30]); the telencephalic vesicles consist of pallial and subpallial territories. (C) Two organizers direct the patterning of the pallium: the hem, at the border of the roof plate and the prospective hippocampus, and the antihem, at the corticostriatal junction near the prospective olfactory cortex ([28]; reviewed in [16]). The morphogen molecules segregated by these organizers form overlapping gradients (solid and dashed arrows) that pattern the pallium in 4 sectors: medial, dorsal, lateral, and ventral pallial sectors. We hypothesize the distinction of 2 parts on the MPall sector corresponding to allocortex (hippocampus) and the adjacent mesocortex (marked with “?”) based on architectonic analysis of adult rats and primates [16]. (D, E) Coronal sections of the adult rat brain (D) and the adult human brain (E) at the level of the anterior commissure show two trends of increasing laminar elaboration according to the Dual Origin of the Neocortex hypothesis ([29]; reviewed in [16]). One trend is traced back to the ancestral hippocampal cortex (solid arrow marks the trend) and the other to the ancestral olfactory cortex (Pir in Panel D, AON in Panel E; dashed arrow marks the trend). The hem and the antihem likely directed the expansion of the neocortex from rodents to primates. ac, anterior commissure; accum, nucleus accumbens; AON, anterior olfactory nucleus in the primary olfactory cortex; cc, corpus callosum; Cd, caudate nucleus; Cl, claustrum; DPall, dorsal pallium; Endo, endopiriform nucleus; LOT, lateral olfactory tract; LPall, lateral pallium; MPall, medial pallium; VPall, ventral pallium; Hipp, anterior extension of the hippocampal formation; Pir, piriform cortex in the primary olfactory cortex; Put, putamen; sm, stria medullaris; TOL, olfactory tubercule.

Systematic variation of laminar structure is also rooted in evolution. Two developmental organizers, the hem and the antihem, have likely directed the expansion of the neocortex from rodents to primates (Fig 3; [28]). The action of these organizers during development is compatible with the Dual Origin of the Neocortex hypothesis, according to which the laminar gradients in the mammalian neocortex can be traced back to the ancestral hippocampal cortex and the ancestral olfactory cortex ([29]; reviewed in [16]). This hypothesis will need to be tested using modern neuroanatomical and molecular methods in future developmental and comparative studies of the hem and the antihem signaling across species including primates.

## Predicting the human cortical connectome and its disruptions in disorders

A key aspect of the Structural Model highlighted by Goulas and colleagues [12] and others [16,20] is that it can be applied to the human cerebral cortex to predict the existence and the laminar pattern of cortico-cortical connections. Moreover, the Structural Model can be used as a framework to compare cortical connections across mammalian species and individual subjects. The Structural Model also provides a basis for the integration of high-resolution neuroanatomical and molecular data with dynamic functional imaging and physiological data to compare neurotypical and pathological conditions in humans.

In line with the predictions of the Structural Model, a recent functional study in human subjects confirmed that cortical areas of comparable architecture are more likely to be connected [31]. With regards to pathology, limbic areas and their feedback pathways are more plastic and more vulnerable to neurological and psychiatric disorders [21], a key feature guiding research in autism [20,32] and proposing testable hypotheses to identify disruption mechanisms in neurodevelopmental disorders [33]. Finally, the Structural Model has been used as the circuit basis for theoretical models of consciousness [6].

## How do chromatin patterns in cortical neurons relate to the connectome?

The emergence of the blueprint of the cortical connectome highlighted by Goulas and colleagues [12] likely depends on epigenetic modifications of cortical neurons during development. The chromatin of early neuroblasts is mostly open, but, as neurons differentiate and connect, genes that are not transcribed are progressively silenced through the formation of closed heterochromatin [33]. This poses an interesting question, relevant for the study of typical and pathological development of the cortex that should be addressed in future research: Are there differences in the time-course of neuron differentiation, pathway formation, and progressive silencing of genes in the nuclei of neurons across developing cortical areas? Can we identify different windows of vulnerability for pathways connecting areas that “develop together and wire together?” Future systematic studies during development in humans and other species across cortical areas with different laminar elaboration may reveal different windows of vulnerability to developmental disruption across the cortical landscape. These studies will have the potential to identify effective interventions to prevent developmental disorders like autism.

In summary, the relationship of cortical structure with the strength and laminar pattern of connections in mammalian species provides a framework to better understand the development and evolution of cortical networks in humans. This framework, validated by Goulas and colleagues [12], can be used in future studies to test hypotheses regarding networks likely affected in neurodevelopmental disorders.

## Abbreviations

SMI-32: Sternberger-Meyer monoclonal antibody to a nonphosphorylated epitope on human NF-M and NF-H subunits of neurofilament protein
